# A Nonlinear Three-Dimensional Finite Element Analysis of Stress Distribution and Microstrain Evaluation in Short Dental Implants with Three Different Implant–Abutment Connections in Single and Splinted Conditions in the Posterior Mandible

**DOI:** 10.1155/2023/8851098

**Published:** 2023-07-27

**Authors:** Karishma S. Talreja, Shobha J. Rodrigues, Umesh Y. Pai, Thilak Shetty, Sharon Saldanha, M. Mahesh, Puneeth Hegde, Satish B. Shenoy, Nithesh Naik, Sandipan Mukherjee, Ann Sales, Vignesh Kamath, Prashant Bajantri

**Affiliations:** ^1^Department of Prosthodontics and Crown and Bridge, Manipal College of Dental Sciences, Mangalore, Manipal Academy of Higher Education, Manipal, Karnataka 576104, India; ^2^Department of Aeronautical and Automobile Engineering, Manipal Institute of Technology, Manipal Academy of Higher Education, Manipal, Karnataka 576104, India; ^3^Department of Mechanical and Industrial Engineering, Manipal Institute of Technology, Manipal Academy of Higher Education, Manipal, Karnataka 576104, India

## Abstract

**Background:**

Stress distribution plays a vital role in the longevity and success of implant-supported prosthesis. This study evaluated the von Mises stress and microstrain in the peri-implant bone and the implant–abutment junction of short dental implants with three different implant–abutment connections in splinted and unsplinted conditions using finite element analysis (FEA).

**Materials and Methods:**

In this experimental study, nine transversely isotropic finite element models were developed, and randomly divided into three equal groups (n = 3): control, (Group AC) single-standard 4.3 × 10 mm bone level implant-supported restorations with external hexagonal (EH) connection, internal conical (IC) and internal trichannel (ITC) connection, single short implant-supported restorations (Group AT), and splinted short implant-supported restorations (Group B) for each of the three implant–abutment connections, respectively. A 200 N load was applied along the long axis of the implants and a 100 N (45°) oblique load was applied and von Mises stress and microstrain values were evaluated.

**Results:**

Single standard implants demonstrated the highest von Mises stress and microstrain values followed by single short implants and splinted short implants, respectively. Among the implant–abutment connections, the IC connection showed the highest values and the ITC connection showed the least values.

**Conclusion:**

Within the limitations of this study, it was concluded that splinting of short dental implants demonstrated lesser and more homogeneous stress and microstrain, especially on oblique loading. The microstrain values for all connections evaluated were within the physiological loading limit (200–2,500 N) and were hence considered safe for clinical use.

## 1. Introduction

The posterior edentulous arches are a biologically and mechanically challenging region for implant-supported rehabilitation. Short dental implants (SDIs) and tilted implants are clinically proven alternatives to complex surgical procedures as they reduce surgical interventions, bring down treatment time and cost, simplify the planning process, and minimize complications [1]. SDI is a sound choice for the replacement of teeth in atrophic arches. SDIs have evolved from short fixtures with narrow diameters and machined surfaces to the current short wide-diameter implants with bioactive surfaces. They are perceived to have a higher failure rate due to their shorter length as compared to standard implants. Studies have shown that the implant neck and the crestal portion of the peri-implant bone (PIB) are subjected to the highest forces and stress concentration. There is a considerable conflict of opinion on the importance of diameter over length or vice versa to minimize stresses in atrophic arches. Implant length may not be a dominant factor as load-bearing forces are more concentrated on the crestal portion of the implant [2].

A cylindrical root form implant 3 mm longer will provide a 20%–30% increase in the surface area, while an increase of 1 mm in the implant diameter increases the functional surface area by 30%–200%, thus improving the load dissipation ability of a wider implant [3]. SDIs are made more promising by the incorporation of surface modifications like acid etching, discrete crystal deposition, titanium blasting, laser ablation, and anodic oxidation that increase the surface area for osseointegration [4]. Much debate exists as to what constitutes the exact length of a short, standard, or long implant [1]. Considering 10 mm as the standard length, an implant less than 10 mm in length is categorized as an SDI and may be used to overcome dimensional limitations in implant placement [2].

PIB loss has a multifactorial etiology and is influenced by patient- and implant-related factors. Load transfer from the implant to the surrounding crestal bone depends primarily on the loading protocol and the implant–abutment connection (IAC) [5]. There is a multitude of IACs in literature, namely, internal hexagonal, external hexagonal (EH), internal octagonal, internal conical (IC), and trilobed. EH, internal hexagonal, and morse taper are the most commonly used IACs. Internal hex incorporates the hex within the implant framework making the implant–abutment system a monobloc, thereby minimizing stress concentration [5]. In external hex connections, stresses induced by masticatory loading are concentrated mainly at the implant–abutment junction (IAJ) primarily due to reduced functional surface area for load transfer [5].

Coppedê et al. [6] correlated the yield strength of the implant–abutment assembly to the prosthetic screw in internal hexagonal connections and IC connections under oblique loading. They concluded that the solid design and friction lock of the IC system gave better strength to the implant–abutment assembly under oblique loads as compared to the internal hex systems. However, studies are needed to assess the extrapolation of these stress patterns in SDIs. Effective lowering of peri-implant stresses has been observed in association with splinted standard implants and is advocated in regions of poor bone density and heavy masticatory force. Similar results with SDIs with internal hexagonal connections have been demonstrated [7, 8]. In addition, internal trichannel (ITC) connection showed lesser equivalent von Mises stress than internal hexagonal and IC connections [9, 10].

A disadvantage noted with SDIs is the increased crown implant ratio [11]. The height of the prosthetic crown is a vertical cantilever that causes an exponential increase in forces with an increase in height. In addition to this angled load, the function is akin to biomechanical force magnifiers thus accelerating crestal bone resorption [12]. Studies have shown that for every 1 mm increase of the crown height space beyond 15 mm, there will be a 20% increase in the stress concentration at the implant crest [13]. In this numerical simulation, we will evaluate the effect of a crown height space of 17 mm on peri-implant stress concentration in SDIs of 8 mm length.

Numerous techniques, including mechanical stress analysis, photoelasticity, and strain measurement, have been used to analyze the stresses on dental structures. However, each of these methods has its drawbacks. Finite element analysis (FEA) is a crucial analytical tool that offers a number of advantages like accurate modeling of complex structures, the ability to change after modeling, and the demonstration of internal stress under different applications of loads by data evaluation within the model [14–16]. However, it is noteworthy to mention that in silico like this, it is not possible to obtain accurate identification of the load that will be transmitted through the crown to the implant and consecutively to the bone, due to the nonfixation of the devices on the surface of the crown or implant, which may result in values lower than those established by literature [17–19].

Most of the recent FEA has obtained their results by assuming isotropic properties of the PIB and 100% bone-implant contact (BIC) [11, 20, 21]. This corresponds to complete osseointegration, which is almost impossible to achieve clinically. BIC varies between 30% and 70% depending on the density of the recipient's bony bed. Block stated that a BIC of 50% correlates to a successful implant. In this study, 50% BIC was chosen as a reasonable parameter to simulate osseointegration in atrophic arches. Bone is a dynamic tissue that is anisotropic, meaning that when its material characteristics are tested in different directions within the same sample, they change. Since the elastic moduli of cortical bone in the buccolingual and inferosuperior directions and cancellous bone in the buccolingual and mesiodistal directions are not considerably different, approximations may be used for computational simplicity. In this study, the mechanical properties of PIB were numerically simulated using the bone's transverse isotropy property.

Meticulous case selection for SDIs can ensure a survival rate that matches implants of standard length. The purpose of this nonlinear three-dimensional (3D) FEA was to predict the stress distribution in three different IACs under axial and oblique loading at an increased crown height space. The study also evaluated the effect of splinting posterior restorations to reduce resorptive stresses on crestal bone in the peri-implant region. The null hypothesis was that the abutment connections, implant splinting, and occlusal load direction have no bearing on the stress and strain distribution.

## 2. Materials and Methods

**Figure 1:**
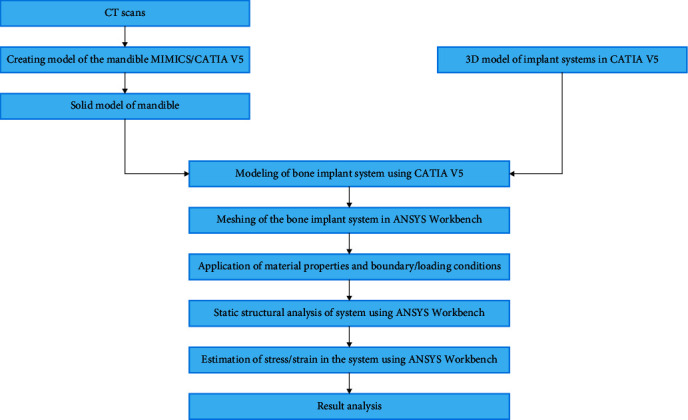
Flowchart of materials and methodology used in the study.

The 3D model is created from Digital Imaging and Communications in Medicine (DICOM) images obtained from cone beam computed tomography (CBCT). This research was conducted with permission from the Manipal Institutional Ethics Committee (MIEC). The CBCT images were obtained from the archives of the Department of Oral Medicine and since it was a retrospective analysis, the ethical committee waived off the need for patient consent. Data acquisition and analysis were performed with the protocols approved by the Institutional Ethical Committee (IEC No. 14126), and the study was performed under ethical standards. All the procedures were performed as per the ethical guidelines laid down by the Declaration of Helsinki (2013). The steps involved in this study and the methodology used are shown in Figure 1.

### 2.1. Creation of Working Models

A 3D surface model was created using MIMICS software (Materialise Interactive Medical Image Control System) using a CBCT image of the edentulous mandible. In order to create a 3D solid model, the surface model was then imported into Computer-Aided 3D Interactive Application (CATIA) software. A 1 mm cortical bone layer was established over the cross-section of the mandible in the second premolar—first molar region and trabecular bone were used in the internal structure to model type III bone. The scanning of representing implants with EH connection (Nobel Speedy Groovy®), IC connection (Nobel Active®), and ITC connection (Nobel Replace Select Tapered™), abutment (Snappy™ Abutment (Nobel Biocare, Switzerland) 5.5, 1.5 mm collar height), abutment screw, supporting splinted (test group), and single crowns (control and test groups were done with a profile projector (Metzer the Profile Projector (METZ-801)) to obtain reverse engineering sketches.

### 2.2. Meshing

**Figure 2:**
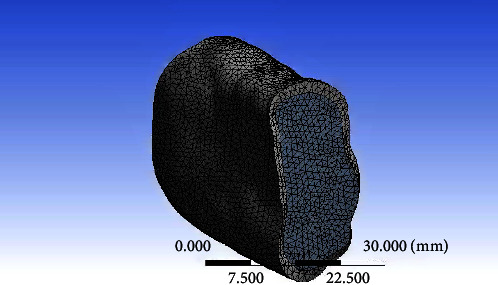
Meshed model of the bone.

The geometric model of the components was obtained using CATIA software. ANSYS 15.0 Workbench software was then used to translate nine 3D finite element models of the implant–abutment restoration complex into the finite element mesh model (Figure 2). A mesh of discrete pieces joined at a limited number of nodes was used to define the entire shape. The initial phase of this analysis involved mesh refining. With the goal of reducing stress fluctuation to under 1%, the average stress in the implant and PIB tissue was calculated using a variety of mesh sizes. The finite element mesh was a 20 node tetrahedron with a mesh size of 0.5 mm. This higher-order element provided a better approximation of complex and curved surfaces of the 3D model. Grid check analysis revealed a convergence of 5% for the chosen mesh size, which resulted in 118,702 nodes and 98,716 elements for the splinted model. To bring the simulation close to the intraoral condition, isotropic properties were applied to the PIB [22].

### 2.3. Boundary Conditions and Material Characteristics

Both the cortical and cancellous bones were thought to be homogeneous, linearly elastic, and isotropic. Ti6Al4V implants were employed in the investigation and the abutments are made of Grade 4 titanium. The control group included implants of standard length (4.3 × 10 mm) and the test group included short implants (5 × 8 mm) for each of the three IACs. The cement-retained restorations included porcelain fused to metal (Wirolloy NB Bego, Germany, and Ceramco®3, Dentsply, US) single crown for an implant in the position of the mandibular right second premolar and splinted crowns for implants in the position of the mandibular right second premolar and first molar. Both restorations were fabricated for a crown height space of 17 mm. Considering 40%–70% of the range for successful osseointegration, a BIC of 50% was used to simulate osseointegration in Type III bone. The abutment and the crown were attached such that there is perfect contact between the abutment screw threads and the implant screw hole. Boundary conditions were modeled to fix the inferior region of the bony tissue and mesial and distal faces of mandibular sections. The boundary conditions between the implant and the bone were set to fixed. The link between the abutment and crown was handled as a contact issue. Therefore, contact analysis (big deflections off) was used in a nonlinear manner.

Tables 1 and 2 enlist the properties of the elements of the finite element mesh as obtained from previous studies [8–10, 22].

### 2.4. Loading Conditions

A vertical 200 *N* load was applied unilaterally in the central fossa region of the right first premolar and the middle of the mesiodistal width of the premolar–molar restoration for the splinted group along with a 100 *N* oblique load at an angle of 45° about the long axis of the implant in a lingual to buccal direction [23–25].

### 2.5. Finite Element Analysis

By utilizing the Ansys 15.0 Workbench software, nonlinear analysis was performed utilizing contact analysis (big deflections off). Number of steps was one, and a step-end time of 1 s was used as the step control. Autotime stepping was controlled by a program. With settings for weak springs controlled by the program, a direct solver type was employed. Inertia relief and large deflections were disabled. The program software automatically regulated nonlinear convergence controls such the Newton–Raphson option, force convergence, moment convergence, displacement convergence, and rotation convergence. For this investigation, the default settings for the variables as listed in the software's help files were used. A nonlinear FEA was used to evaluate the von Mises stress at the IAJ and PIB. Microstrain values in PIB were also evaluated. Three factors were studied: connection type (IC, ITC, and EH); the effect of splinting (splinted and nonsplinted restorations); and the direction of occlusal load (axial and oblique).

## 3. Results

### 3.1. Stress and Strain in the Peri-Implant Bone

**Figure 3:**
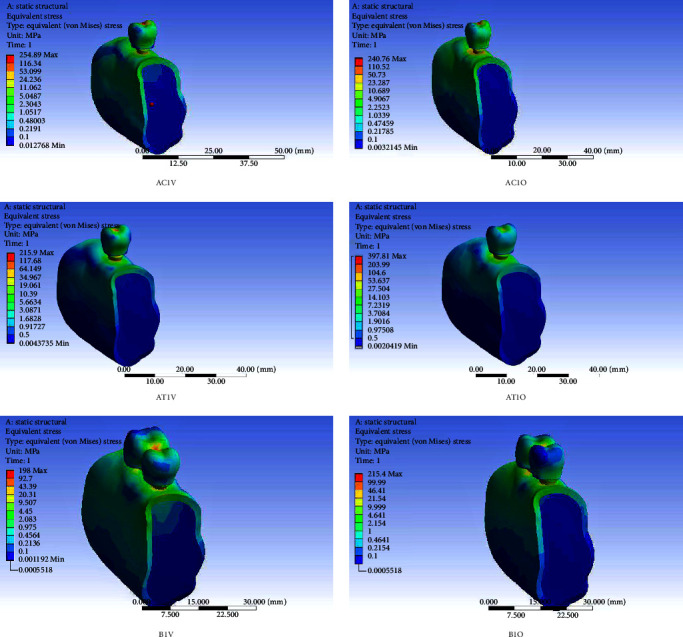
von Mises stress in peri-implant bone in finite element models of a single-standard implant-supported crown (AC), a single short implant-supported crown (AT), and short implant-supported splinted restorations (B) with the external hexagonal connection (AC1V, AC1O, AT1V, AT1O, B1V, and B1O) under vertical (V) and oblique (O) loading.

**Figure 4:**
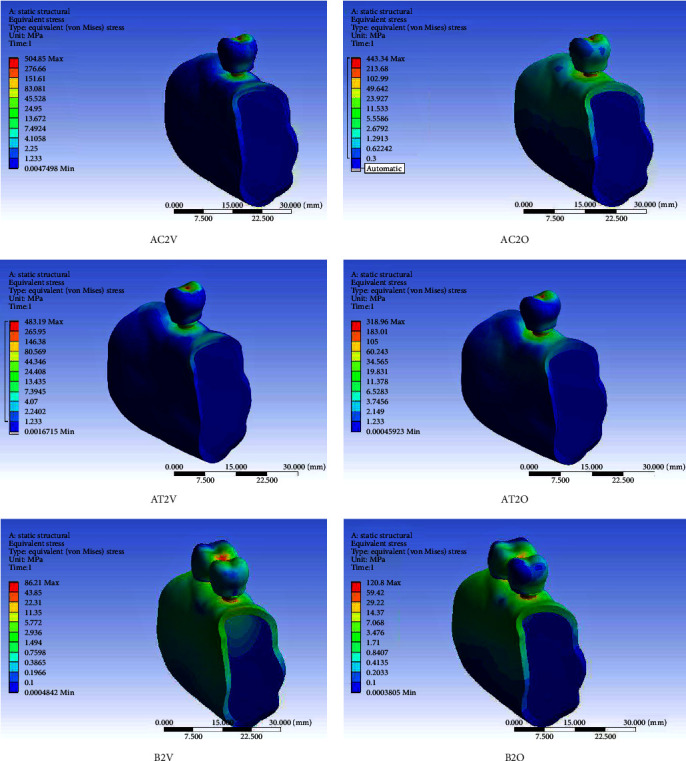
von Mises stress in peri-implant bone in finite element models of the single-standard implant-supported crown (AC), a single short implant-supported crown (AT), and short implant-supported splinted restorations (B) with the internal conical connection (AC2V, AC2O, AT2V, AT2O, B2V, and B2O) under vertical (V) and oblique (O) loading.

**Figure 5:**
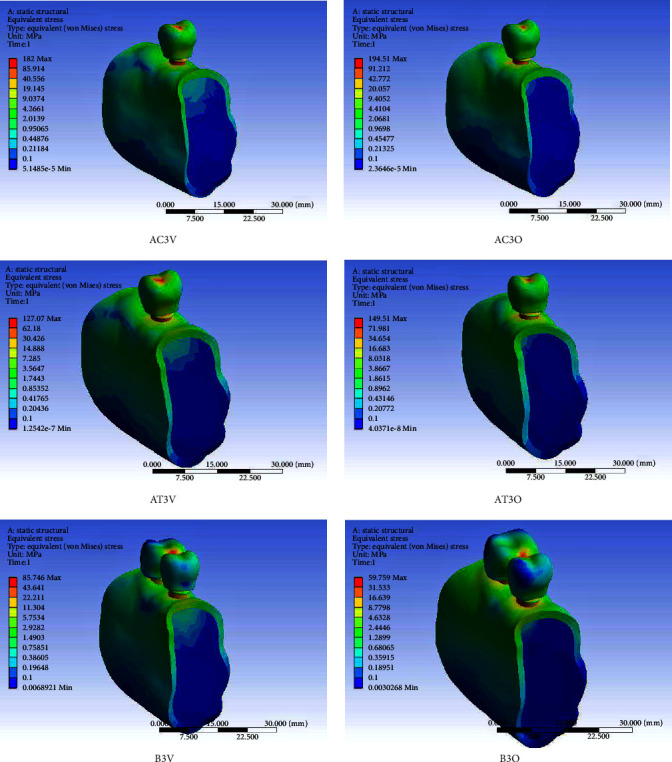
von Mises stress in peri-implant bone in finite element models of the single-standard implant-supported crown (AC), a single short implant-supported crown (AT), and short implant-supported splinted restorations (B) with the internal trichannel connection (AC3V, AC3O, AT3V, AT3O, B3V, and B3O) under vertical (V) and oblique (O) loading.

Short dental implant-supported restorations demonstrated lower von Mises stress and microstrain in the PIB when compared to the control group (standard implants) under both axial and oblique loading and for all three IACs. On vertical loading, there was a 52.42% reduction in stress in PIB in EH, 30.86% in IC, and 64.92% in ITC in comparison to similar connections in implants of standard length. On oblique loading, there was 20.44%, 28.28%, and 15.96% reduction in the three abutment connections, respectively, as compared to similar connections in standard implants (Tables 3 and 4). Splinting of SDIs led to a definite lowering of stress and microstrain in PIB irrespective of the IAC, especially on oblique loading. In the context of the type of IAC, the ITC connection caused the least stress concentration in the PIB followed by the EH and the IC connections. On axially loaded splinted implants, the von Mises stress recorded was 1.6 times lesser than those recorded on loading single simple implant restorations for all the three tested IAC. On oblique-loaded splinted implants, a reduction of 2.86, 1.90, and 2.17 times was observed in EH, IC, and ITC connections, respectively. The microstrain values recorded in splinted short implants were lowest in comparison to the corresponding connections in single-standard and short implant-supported restorations. In addition, the microstrain values in splinted short implants were lower on oblique loading than vertical loading in the corresponding connections. In comparison to single short implant-supported restorations, the microstrain values on oblique loading in splinted short implants were 2.86 times lesser in EH, 1.90 times lesser in IC, and 2.16 times lesser in the ITC connections. In the splinted SDIs, the ITC connection on vertical loading showed the lowest microstrain of all the nine models (245 *µε*) while the IC on oblique loading showed the highest microstrain (856.75 *µε*). Figures 3–5 show the von Mises stress in PIB in the finite element models considered in the study.

#### 3.1.1. Stresses at the Implant–Abutment Junction

Like the PIB, von Mises stress at the IAJ for splinted and single short implants was lower than their counterparts of standard length (Table 5). The von Mises stress observed in all three IACs tested was higher for oblique loading. SDIs reduced von Mises stress by 49.68% in EH, 32.46% in IC, and 62.32% in ITC connections on vertical loading as compared to single crowns supported by standard implants. Increased stress concentration on the buccal aspect was noted in all three IACs. The stress concentration was most pronounced on the buccal aspect of the abutment collar of the ITC connection though it showed the least numerical value. The EH connection showed an even distribution of stress at the implant–abutment interface. The reduction of stresses in comparison to standard implants on oblique loading was not as appreciable as the reduction in stresses noted on vertical loading. On oblique loading, EH connections reduced stresses by 10.77%, IC by 36.45%, and ITC connection by 17.25% as compared to standard implants. The IC showed the highest von Mises stresses followed by the EH and the ITC connections that showed the lowest stresses. SDIs demonstrated a marked rise in the von Mises stress at the implant–abutment interface when loading was changed from vertical to oblique direction. EH connections recorded an increase of 143.19% in the von Mises stress, which was highest when compared to the IC (80.37%) and ITC connections (70.56%). SDIs with EH and ITC connections caused a greater percent increase in the stresses at the implant–abutment interface as compared to the control groups with similar connections. On the other hand, the percent increase in the stresses from oblique to vertical loading was comparable at the implant–abutment interface of IC-connected short (80.37%) and standard (85.8%) implants. Splinting had a positive influence on the reduction of von Mises stress at the implant–abutment interface on both axial and oblique loading. Splinted implants recorded stresses that were 2.92 times lesser in EH, 1.91 times lesser in IC, and 2.12 times lesser in ITC connections compared to single short implants. Splinted implants with ITC connections showed the lowest stresses at the implant–abutment interface among the nine 3D models evaluated, while standard implants with IC connections showed the highest stresses under both vertical and oblique loading.

## 4. Discussion

The null hypothesis was rejected since stress and strain distribution was influenced by the choice of abutment connections, splinting of implants, and direction of occlusal load. In recent years, various methods have been put forth to avoid invasive surgical augmentation treatments while predictably rehabilitating patients with atrophic residual bone [26–28]. Tilted and SDIs were developed to facilitate a wrinkle-free treatment plan for such patients with atrophic ridges. Tilted implants have been particularly introduced in clinical practice, to preserve anatomical structures, such as the maxillary sinus and the mandibular nerve [29, 30].

In full-arch immediate loading rehabilitation, when longer implants are favored to increase primary stability since there is less bone in the distal sites, this approach is especially advised [29].

SDIs have evolved from short fixtures with narrow diameters and machined surfaces to the current short wide-diameter implants with bioactive surfaces. They are perceived to have a higher failure rate due to their shorter length as compared to standard implants. Studies have shown that the implant neck and the crestal portion of the PIB are subjected to the highest forces and stress concentration. There is a considerable conflict of opinion on the importance of diameter over length or vice versa to minimize stresses in atrophic arches.

In all IACs, for all lengths of the implants, in both splinted and single restorations, the von Mises stress and microstrain recorded on oblique loading were higher than those recorded on vertical loading. These results were in concurrence with observations made in the literature. de Vasconcellos et al. [31] noticed higher values of microstrain when implant-supported prostheses are nonaxially loaded in comparison to axial loading. A direct proportionality between the von Mises stress and microstrain has been observed and hence both parameters are discussed together.

While the stress and strain observed in SDIs were lower than those observed in standard implants, the percentage increase in the von Mises stress and microstrain from vertical to oblique loading were higher in short implants. While implants of standard length noted an increase of 88.5%, 82.2%, and 26.38% from vertical to oblique loading with EH, IC, and ITC, respectively, a corresponding increase of 138.6%, 85.89%, and 79.7%, respectively, was noted in SDIs. It was concluded that a sharp rise in the peri-implant stresses and microstrain may be observed during chewing when the patient shifts from centric to eccentric positions and likewise during parafunctional activities in short dental implant-supported unsplinted prostheses.

There was a significant reduction in von Mises stress and microstrain in both PIB and the IAJ when comparing short implants to standard implants. In addition, splinting resulted in a reduction of stresses and microstrain in both axial and oblique loading in all three IACs. On axial loading, the von Mises stress and microstrain in the PIB of splinted implants were 1.5–1.6 times lesser than single short implants, while a reduction of 1.9–2.9 times was observed on oblique loading. Therefore, splinting of SDI-supported prostheses may be prudent as it will brace the units against detrimental forces and minimize bone loss on account of better force dissipation, especially on oblique loading. The results of this study are in agreement with similar studies done by Quaranta et al. [32], Toniollo et al. [20], Yang et al. [7], Kim et al. [11], Fugazzotto [33], and Anitua et al. [34]. Another noteworthy fact is that in our study, the implant diameter for SDIs was 5 mm, which is more than the diameter evaluated by Quaranta et al. [32].

According to a FEM simulation by Anitua et al. [34], increased implant diameter may reduce the maximum von Mises stress in PIB by 20%–30%. Additionally, this study demonstrated that occlusal forces are mostly centered at the implant's first three threads and considerably diminish after these threads. The stress around the implant neck, however, lessens as implant diameter rises (in implants with a 5 or 5.5 mm diameter), and it is better dispersed along the bone-implant contact. As a result, Anitua and colleagues believed that the impact of implant diameter on bone stress distribution was more substantial than the impact of the implant's length or geometry. The results of this study are not in agreement with similar studies done by Misch who recommends that 8 mm should be the recommended height for dense bone and increase progressively as bone quality decreases [3].

One of the most criticized aspects of the use of short implants is their unfavorable crown-to-implant ratio (C/I). Normal teeth have a fulcrum that shifts apically as the bone gradually resorbs, making them more susceptible to lateral occlusal forces that might cause damage. Misch [35] contended that because osseointegrated dental implants are ankylosed to bone and lack a center of rotation two-thirds of the way down the endosteal/root portion, their length is not directly related to their ability to withstand lateral forces and should not be used as a predictor of implant survival. As previously mentioned, the initial coronal threads of osseointegrated implants are where the majority of occlusal stresses are concentrated. These results support splinting of short implants for better force dissipation under oblique loading and to minimize bone loss. For all the models evaluated, the IC connection showed maximum von Mises stress and microstrain in both the IAJ and the PIB. All three IACs evaluated showed an even distribution of stresses in the PIB on axial loading. While the IC connection showed an even pattern of distribution of oblique loads in the PIB, the EH connection showed a region of stress concentration buccally, which may lead to bone resorption. The differences in the stresses in the PIB were the results of the differences in the load transfer mechanism of the different abutment connections. The EH implant has a butt joint and most of the stresses are taken up by the cortical bone adjacent to the first thread leading to areas of stress concentration. The high stresses seen in the PIB of implants with IC connections are in agreement with similar studies done by Chun et al. [36] but in contrast to the results of Sarfaraz et al. [37]. However, in this study, the IC connection simulated is one with an inherent platform switch and has a crown height space of 17 mm. These factors may have amounted to the stress patterns observed.

The stress distribution at the IAJ in EH connections was uniform under vertical and oblique loading in both single and splinted short implants but showed a concentration of stress on the buccal side for standard implants when loaded obliquely. This may be attributed to the larger area available for dissipation of stresses in short wide implants The internal connections amounted to an area of stress concentration on the buccal and lingual aspect on oblique loading in standard, single short, and splinted short implant groups. The location of these stresses was at the abutment collar and showed a pattern similar to that observed by Lan et al. [38].

The height of the abutment collar chosen for this study was 1.5 mm, which is more than the usually simulated collar height (0.5 mm). This increase may have increased the vertical cantilever on the implant–abutment interface and amounted to the red zones on the color maps. This area of stress concentration dulled out on splinting short implants with the ITC connection. These observations added to the advantages of splinting short implants in the ITC group.

The EH connection has not been compared to the IC connection in literature; however, there is a general trend observed that IC connections show lesser von Mises stress compared to EH connections. Lan et al. [38] found higher stresses in ITC connections, which is in contrast with our study. The higher stresses in the IC connection may be due to one of many factors like the different implant geometries modeled for this study, anisotropic properties of modeled bone, inherent platform shift in the Nobel Active® design, or the increased crown height space in this study. Pozzi et al. [39] in a randomized controlled trial noted a significantly lesser bone loss for Nobel Active® implants (IC) after 3 years of function as compared to the Nobel Speedy Groovy® design (EH), while Rokn et al. [40] found no statistically significant difference in the two designs up to 1 year of function. The lack of bone loss seen in the Nobel Active® implant may be due to its superior implant surface modifications and thread geometry.

IC connections have shown the highest von Mises stress and microstrain at the IAJ and PIB. Also, the stresses seen in splinted implants with IC connections are comparable to standard implants with ITC connections, which generate the least stress. Hence, the study recommends splinting of SDI with IC connections. The use of sample size (*n* = 1) in this study is substantiated by the convergence of the grid check performed for both single and splinted groups. All the von Mises stress values obtained for the test groups were lower than the corresponding values of standard implants with similar IACs.

According to Frost's mechanostat theory if the microstrain in PIB is below 200 *µε*, the bone undergoes disuse atrophy and if it crosses 2,500 *µε*, hypertrophy followed by bone resorption occurs [41]. The microstrain values for all the models evaluated under both vertical and oblique loading were within the limit for physiological loading of bone (2,500 *µε*). The criteria for success applied to standard implants may not carry the same significance for SDIs. Keeping this in mind, the practitioner needs to understand that a comparison between implants placed in surgically enhanced or augmented sites and SDIs may be more useful to draw clinical inferences than one between standard and short implants.

The study being discussed evaluated the biomechanical performance of different IACs in SDIs under masticatory loads. However, the study had some limitations, including the inability of FEA to fully mimic the geometric response of bone to masticatory loads and the static simulation of a dynamic process like mastication. These limitations restricted the FEA to a more qualitative analysis rather than a quantitative one. Moreover, the study did not take into account the effects of the temporomandibular joint and masticatory muscles on the IAC. Despite these limitations, the study found no biomechanical drawbacks to the clinical usage of any of the three implant–abutment designs tested. This suggests that all three designs can be safely used in SDIs. However, the author suggests that further research could focus on the effect of different IACs on immediate loading, immediate placement of short implants, and cantilevered restorations to SDIs.

In summary, while the study had some limitations, it provides useful insights into the biomechanical performance of different IACs in SDIs. Future research can build on this work to investigate the effects of other factors on IACs and to improve our understanding of the biomechanics of dental implants. Ultimately, this knowledge can help to improve the safety and efficacy of dental implant procedures and enhance patient outcomes [42].

## 5. Conclusion

The biomechanical performance of various IACs in SDIs under masticatory loads was investigated in this study. The study's conclusions can help clinicians choose the best IACs and restorative solutions. The study's use of computer models rather than clinical data, however, places certain restrictions on it. To verify the study's findings and determine their therapeutic relevance, more investigation is required. The study discovered that, compared to axial loading, oblique loading produced increased von Mises stress and microstrain at the IAJ and PIB. Furthermore, compared to long implant-supported single restorations, short implant-supported single restorations showed decreased von Mises stress and microstrain. In addition, the study showed that splinting SDIs had a favorable impact on reducing stresses and microstrain in SDI-supported restorations, which may help reduce bone loss under oblique loading.

Additionally, among the connections examined, ITC connections, followed by EH and IC connections, recorded the lowest stress and microstrain values. All models tested under vertical and oblique loading had microstrain values that were within the range for physiological loading of bone, showing that the models were not overloading the bone. In conclusion, this research sheds important light on how various implant–abutment relationships behave biomechanically in SDIs. However, additional study is required to verify the study's findings and determine their therapeutic applicability. Future research should investigate additional variables that might have an impact on how well implant–abutment linkages and restorative solutions function biomechanically. In the end, a better comprehension of the biomechanics of dental implants can result in safer and more efficient dental implant treatments as well as better patient results.

## Figures and Tables

**Table 1:** Material properties of bone.

Properties	Cancellous bone	Cortical bone	Reference
Youngs modulus *E* (MPa)	1,148	19,400	Kurniawan et al. [22]
Poisson's ratio *ν*	0.32	0.3	Kurniawan et al. [22]

**Table 2:** Mechanical properties of materials.

Structural element	Poisson's ratio *ν*	Young's modulus (GPa)	Reference
Ti6Al4V (implant and abutment)	0.35	110	Sertgöz [23]
Cement layer	0.35	22.4	Anusavice and Hojjatie [24]
Ni–Cr alloy	0.33	206.6	Anusavice and Hojjatie [24]
Feldspathic porcelain	0.35	82.8	Eskitascioglu et al. [25]

**Table 3:** von Mises stress values (MPa) in the peri-implant bone for standard, short, and splinted short implant-supported restorations with the different connections evaluated under vertical and oblique loading.

	EH	IC	ITC
Standard implant (Group AC)			
Vertical loading	90.10	121.72	72.12
Oblique loading	169.91	221.81	91.15
Single short implant (Group AT)			
Vertical loading	59.11	93.01	43.73
Oblique loading	141.07	172.90	78.60
Splinted short implants (Group B)			
Vertical loading	35.143	59.32	26.05
Oblique loading	49.25	90.89	36.18

**Table 4:** Microstrain values in peri-implant bone for standard, short, and splinted short implant-supported restorations with the different connections evaluated under vertical and oblique loading.

	EH	IC	ITC
Standard implant (Group AC)			
Vertical loading	849.34	1,147.36	679.88
Oblique loading	1,601.61	2,090.83	863.89
Single short implant (Group AT)			
Vertical loading	557.16	876.76	412.16
Oblique loading	1,329.75	1,629.79	740.09
Splinted short implant (Group B)			
Vertical loading	331.26	559.14	245.57
Oblique loading	464.23	856.75	341.06

**Table 5:** von Mises stress values (MPa) at the implant–abutment junction for standard, short, and splinted short implant-supported restorations with the different connections evaluated under vertical and oblique loading.

	EH	IC	ITC
Standard implant (Group AC)			
Vertical loading	98.58	136.35	79.81
Oblique loading	177.43	253.34	98.38
Single short implant (Group AT)			
Vertical loading	65.86	102.93	49.19
Oblique loading	160.17	185.66	83.90
Splinted short implant (Group B)			
Vertical loading	40.24	66.85	30.25
Oblique loading	54.79	97.1	39.48

## Data Availability

Data will be provided by the corresponding author or first author on reasonable request.
